# Preclinical Models for Cryptococcosis of the CNS and Their Characterization Using In Vivo Imaging Techniques

**DOI:** 10.3390/jof10020146

**Published:** 2024-02-12

**Authors:** Lara Roosen, Dries Maes, Luigi Musetta, Uwe Himmelreich

**Affiliations:** Biomedical MRI, Department of Imaging and Pathology, KU Leuven, 3000 Leuven, Belgium; lara.roosen@kuleuven.be (L.R.); dries.maes@kuleuven.be (D.M.); luigi.musetta@kuleuven.be (L.M.)

**Keywords:** *Cryptococcus*, in vivo imaging, preclinical models, central nervous system, MRI, CT, BLI, FLI, multiphoton microscopy

## Abstract

Infections caused by *Cryptococcus neoformans* and *Cryptococcus gattii* remain a challenge to our healthcare systems as they are still difficult to treat. In order to improve treatment success, in particular for infections that have disseminated to the central nervous system, a better understanding of the disease is needed, addressing questions like how it evolves from a pulmonary to a brain disease and how novel treatment approaches can be developed and validated. This requires not only clinical research and research on the microorganisms in a laboratory environment but also preclinical models in order to study cryptococci in the host. We provide an overview of available preclinical models, with particular emphasis on models of cryptococcosis in rodents. In order to further improve the characterization of rodent models, in particular the dynamic aspects of disease manifestation, development, and ultimate treatment, preclinical in vivo imaging methods are increasingly used, mainly in research for oncological, neurological, and cardiac diseases. In vivo imaging applications for fungal infections are rather sparse. A second aspect of this review is how research on models of cryptococcosis can benefit from in vivo imaging methods that not only provide information on morphology and tissue structure but also on function, metabolism, and cellular properties in a non-invasive way.

## 1. Introduction

Of the more than six million known fungal species, only 1% have been reported to cause infections in humans [1]. These fungal pathogens are a continuing threat due to the ever-expanding use of immunosuppressants and the increasing number of immunocompromised patients [2,3]. In addition, there is a rise in resistance to often outdated antifungal treatments [1,4,5]. To raise awareness, the World Health Organization published four critical groups, namely *Candida albicans*, *Candida auris*, *Aspergillus fumigatus*, and foremost, the *Cryptococcus neoformans*/*Cryptococcus gattii* (CN/CG) species complexes [6]. While estimations of the global burden of CN/CG infections are difficult, as outlined by Kabir and Cunningham (see [7] and references therein), the number of global infections may reach up to one million cases yearly with still poor survival rates. Only a few cases of CN are reported in immunocompetent patients, while CG more frequently emerges in healthy individuals [8,9,10]. In the 1950s, there were fewer than 300 reported cryptococcosis cases globally [11]. This number of cases increased significantly in the following years, primarily due to the rising prevalence of AIDS combined with more widely available diagnostic toolkits [8,12,13,14]. The inhalation of the basidiospores and desiccated yeasts from their environmental source may cause either an asymptomatic exposure or, in rare instances, a symptomatic self-resolving pulmonary infection [15,16].

**Figure 1:**
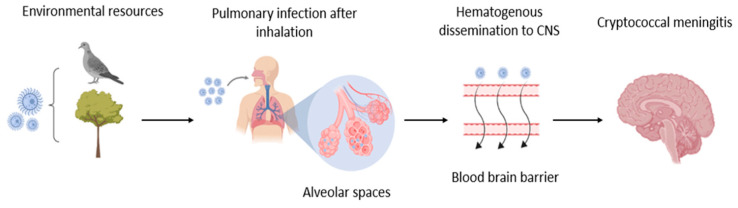
Schematic representation of the pathogenesis of cryptococcosis. (1) CN and CG are present in the environment (for example, in pigeon guano and in trees). (2) The particles (yeast cells or spores) are inhaled by a person. (3) The portal entry is the lungs, where the particles can establish a lung infection if the immune response fails to clear them. (4) When escaping the lungs, cryptococcosis can disseminate to other organs via the bloodstream. (5) Due to the neurotropism of the fungi, the CNS is subsequently infected. This figure was adapted from Bermas et al. [53].

Concerning immunocompromised patients, the inhaled cryptococci (1–5 µm) deposit in the pulmonary alveoli results in non-specific pulmonary manifestations such as dyspnea, cough, and fever [17,18]. If left untreated, this pulmonary infection may evolve into systemic cryptococcosis as a consequence of hematogenous dissemination [9,19,20]. During the initial pulmonary stage of the infection, the adaptive and innate immune systems are activated, in which the alveolar macrophages play a decisive role due to their ability to phagocyte fungal cells [21,22,23,24]. After the initial immune response, the cryptococci are cleared effectively by the adaptive immune system (CD4^+^, CD8^+^, macrophages) with only minimal inflammatory reactions [21,22,25,26,27]. In other instances, the fungi transition into a latent stage, remaining dormant for years [9,28]. When the host mechanism is compromised, reactivation may be provoked, leading to a disseminated outbreak [29]. The fungal cells have the ability to escape the lung alveoli, affecting other organs such as the skin, myocardium, bones, joints, urinary tract, and prostate, eliciting non-specific symptoms such as fever, chest pain, weight loss, cough, and respiratory distress [30]. This often complicates a proper diagnosis and the subsequent start of a suitable course of treatment [25,31]. In the final phase of the infection, the cryptococci exhibit neurotropism towards the central nervous system (CNS) [30,32,33,34]. After crossing the blood–brain barrier (BBB), cryptococcal meningoencephalitis (CME) and/or cerebral mass lesion formation, called cryptococcomas, occurs [1,35,36,37,38]. At this stage, the patients exhibit neurological sequelae such as, but not limited to, fever, altered mental status, meningeal irritation, headaches, lethargy, coma, and eventually death [1,39,40] (Figure 1). 

Since establishing a correct diagnosis for cryptococcosis is complex, a combination of assessing symptoms, imaging tools, and advanced molecular microbiological laboratory tools is necessary for confirmation. The latter consists of culturing, histopathology, molecular detection, serology, and microscopy ([9,12,41,42]. The golden standard for the diagnosis of cryptococcosis is based on the culturing of the yeast from body fluid (cerebrospinal fluid (CSF)) or tissue biopsies [9,39,43]. To date, therapeutic options without severe side effects are limited [5,44,45]. To devise optimal treatment strategies, it is imperative to elucidate the underlying biological mechanisms of cryptococcosis and investigate the host–pathogen interactions prior to and during the infection. This, in turn, plays a crucial role in establishing a diagnosis, treatment opportunities, and prevention methods [5,46,47]. 

In order to test hypotheses on the pathogenesis and dissemination of cryptococcosis and to evaluate novel therapeutic approaches, vertebrate animal models are essential [32,48,49,50,51,52]. Because of the dynamic processes involved, it is imperative to not only use conventional invasive methods for their characterization but to develop longitudinal, non-invasive strategies to elucidate the underlying factors at play. 

Here, we provide an overview of animal models for cryptococcosis, with a focus on rodents. In addition, we elaborate on the potential of in vivo imaging tools to reliably follow up on pathogenesis and therapy in a preclinical context.

### Data Collection

A thorough and comprehensive search was conducted for cases documented in the English literature by querying the ‘PubMed’ and ‘Web of Science’ databases. The search utilized terms such as ‘*Cryptococcus*’, ‘*Cryptococcus neoformans*’, ‘*Cryptococcus gattii*’, ‘Fungus’, ‘Infection’, ‘Imaging’, ‘Multimodal imaging’, ‘rodent model’, ‘murine model’, ‘Cryptococcal models’, ‘Cryptococcal inoculation routes’ and ‘cryptococcosis’. 

## 2. Non-Mammalian Models

### 2.1. In Vitro Models

CN and CG have been long-established model organisms within the field of medical mycology [54,55]. It is often constituted as a basis for (in vitro) studies concerning antifungal drug testing, drug resistance, mechanisms behind chronic infections and radioimmunotherapy, fungal growth, and environmental interactions [56,57,58,59]. Despite the frequent use of CN/CG in preclinical research, there are still gaps in our knowledge about the mechanisms that drive cryptococcal infection and dissemination. One of the focal points is how relatively large cells such as cryptococci are able to surpass natural barriers like the BBB, a crucial step in the disease’s progression [60,61,62]. Though several theories exist, the arguably most remarkable theory on how fungal cells may transverse the BBB is dubbed the “Trojan Horse Hypothesis” [63,64]. This framework suggests the involvement of alveolar M2 macrophages as a carrier host cell for cryptococci to surpass several physiological barriers [1,61,63,65]. Additionally, two other theories described in the literature are transcytosis and paracytosis (see below). 

Some in vitro observations of this event have been made using 3D BBB model chambers in strictly controlled environments, though it has yet to be confirmed within an in vivo disease model [63]. While in vitro models are very helpful in testing basic concepts, it is virtually improbable to fully model the complex host–pathogen interactions [25,66,67]. 

### 2.2. Invertebrate Models

Introducing animal models to preclinical research may resolve the majority of hurdles inherent to in vitro settings, as described a priori. Several invertebrate organisms have served as valuable models for the study of cryptococcosis. These include *Caenorhabditis elegans*, *Drosophila melanogaster*, amoeboid models, *Galleria mellonella*, and *Bombyx mori* [48,68,69,70,71,72,73]. Invertebrate model systems enable relatively high-throughput screening of antifungal compounds and identification of virulence factors [70,74,75]. The primary advantages lie in their short reproduction times, limited ethical concerns, and lower costs when compared to their mammalian counterparts [68]. However, it is important to note that these models lack an adaptive immune system, skewing the normal disease progression [76]. Despite this, they still offer valuable insights into evolutionarily conserved mechanisms of the innate host defense that have been proven to initiate the principal antifungal response during infection [77,78,79].

The majority of insect models function as the basis for proof-of-principle studies concerning antifungal therapies and basic host–pathogen interactions before moving to more sophisticated vertebrate models, in which the disease progression, as reported in humans, is more easily reproduced. A more comprehensive review of the utilization of invertebrate models for studying cryptococcosis in vivo has been elucidated in the review articles by Normile et al. in 2020 and Sabitii et al. in 2012 [48,80].

## 3. Vertebrate Models

To study the pathogenesis of CN/CG, several model systems exist to mimic human cryptococcosis, with respective advantages and disadvantages. However, no model is fully representative of cryptococcal infections in humans [80]. The use of vertebrate models offers a strategic solution to surmount the challenges posed by in vitro models and invertebrate models, as these vertebrate counterparts more closely emulate the pathogenesis observed in humans [81,82,83]. Mammalian models, in particular, have played a pivotal role in enhancing our comprehension of the interaction dynamics between cryptococci and the host’s environment [84,85]. This evolution is notably evident within the context of longitudinal follow-up studies [86]. Moreover, the application of vertebrate models offers a strategic solution to surmount the challenges posed by in vitro models, as these vertebrate counterparts more closely emulate the pathogenesis observed in humans [48,50,52,80]. These models significantly overcome limitations for understanding the progression of cryptococcal infection, including the immune response and the dissemination from the lungs to other organs, notably the brain [87,88,89]. It is more practical to collect larger tissue and body fluid samples, such as biopsies, blood, or CSF, repeatedly, which would otherwise be challenging in invertebrates [90,91]. 

The most frequently used animal model to study fungal infections is inbred mice (*Mus musculus*), though other species are also used, such as rats (*Rattus norvegicus*), guinea pigs (*Cavia porcellus*), rabbits (*Oryctolagus cuniculus*), and zebrafish (*Danio rerio*). For models in rats and mice, Table 1 provides a selection of studies and their main purpose.

### 3.1. Mus musculus (Mice)

Cryptococcosis has been extensively researched in mice due to their high susceptibility to various strains of CN [92,110]. The murine model is often chosen to assess fungal infections because it is a well-established and characterized model with high similarities compared to humans regarding similar organ systems and 99% of gene similarities [48,111]. This model offers the advantages of diverse genetic backgrounds (including those with a modified immune system) and cost-effectiveness in procurement and housing. Infection can be induced through various routes: intranasal inhalation (INI), intratracheal (ITI) administration, intravenous injection (IVI), intraperitoneal injection (IPI), stereotactic injection (STI), intracisternal injection (ICI), and cutaneous inoculation (see below) [62,91,112]. Additional advantages are the relatively low maintenance costs, the ease of handling mice, and the availability of many inbred and transgenic mouse strains [48]. Past studies have shown differences between inbred strains in survival rates, inoculum doses (even when administered via the same route), yielding differences in susceptibility, and disease progression [93,94,95]. C57BL/6, A/J, CBA/J, DBA/J, and BALB/c mouse strains are most often used to study experimental *Cryptococcus* infection models [96,97,98]. A study by Davis et al. indicated discrepancies between the C57BJ/6J, FVB/J, and SJL/J strains observed due to differences in immune response [95]. Another study performed by Zaragoza et al. observed immunological differences in the susceptibility of CBA/J and BALB/C strains when injected with IT but not when injected with IV [94]. 

### 3.2. Rattus norvegicus (Rat)

Utilizing rat models presents several advantages compared to their mouse counterparts: their size allows for various manipulations, including endotracheal intubation, bronchoalveolar lavage, sequential venepuncture, and CSF collection [90,91]. The typical host response of rats to infection has been comprehensively studied following ITI using a well-characterized CN profile [103,104]. Krockenberger and colleagues established a pulmonary cryptococcosis model in rats by the instillation of four molecular types of CG [90]. It was observed that pulmonary cryptococcosis caused by CG in rats is distinct from that of CN, with progressive and ultimately fatal infections [90]. 

Metabolism of the pathogen and potential differential diagnosis have been studied in pulmonary and cerebral cryptococcosis in rats [105,106]. While rats serve as an established and robust model species for cryptococcosis, the drawbacks of this model include the increased cost and the sparsity of genetically modified variants for investigating the impact of host parameters on cryptococcosis [80]. 

Despite the natural prevalence and natural resistance of the disease in rats, only limited research has been conducted in the past using rat models [4,48,49,90,107,113,114,115]. Many studies on cryptococcosis in rat models commonly emphasize the pulmonary dimension, with Graybill’s research group being among the pioneers in assessing pulmonary cryptococcosis in these models [107]. More recently, rat models have increasingly been used to study the treatment of cryptococcosis. For example, in a study performed by Staudt et al., a rat model was used to study the effect of voriconazole (a triazole antifungal) to treat cryptococcal meningitis caused by CN [116].

### 3.3. Cavia porcellus (Guinea pig)

Guinea pigs are a popular model to study fungal infections like cutaneous *C. albicans* infections, systemic aspergillosis, and cryptococcosis [117,118,119]. In general, guinea pigs have a tame nature and are of medium size, as well as being generally susceptible to fungal infections [120]. However, a drawback related to using guinea pigs in research is their intricate social hierarchy, which makes them susceptible to stress when exposed to unfamiliar surroundings, housing arrangements, or the necessary procedures during experimentation [48,121]. Additionally, the costs associated with their upkeep and housing are relatively high compared to most of the other mammalian models listed here. Most guinea pig studies regarding experimental cryptococcosis were performed between the 1970s and 1990s. An example of such a study is the one performed by Riera et al., where guinea pigs were infected, IP with CN, and the formation of lesions in the lungs, liver, spleen, and brain was monitored while studying the humoral immune response [122]. Later on, this guinea pig model was used to study cryptococcal meningitis by Kirkpatrick et al. in order to evaluate the efficacy of antifungal drugs [123]. More recent studies include one by Cai et al. This research group infected guinea pigs subcutaneously with CN to characterize the antigenicity of Cpl1 [124].

### 3.4. Oryctolagus cuniculus (Rabbit)

In the past, rabbits have not been commonly utilized as hosts for studying cryptococcosis due to the associated high costs of acquisition and husbandry. However, despite their inherent resistance to cryptococcal infections and being a corticosteroid-sensitive species, rabbits are nowadays used as a model to examine disease progression [90,125]. Previous studies showed their tolerance to IVI and IPI injections of a large inoculum of CN without developing a lasting or fatal infection [90,126]. Felton et al. observed pulmonary lesions post-intratracheal injection in rabbits, noting the absence of cryptococcal dissemination in the CNS [127]. The rabbit’s elevated body temperature, inhibiting fungal growth, may contribute to their natural resistance [126,128]. 

It has to be noted that the needed corticosteroid treatment alters cryptococcal infection progression significantly but may also act as a model for respective immunocompromised patients [90,126,128]. Historically, rabbits were popular to study the proliferation stage during CNS infections like cryptococcal meningitis, together with treatment strategies against meningitis [128,129,130,131]. 

### 3.5. Dario rerio (Zebrafish)

Zebrafish models are gaining prominence as an alternative and cost-effective model for studying CNS pathologies, offering easy mutagenesis and optical transparency [132,133]. It mimics human immune responses to fungal infections and poses fewer ethical concerns [134,135,136,137]. Zebrafish serve as a compromise between invertebrate simplicity and mammalian organ complexity. Studies on cryptococcosis in zebrafish reveal insights into host–pathogen interactions [133,138]. Cutting-edge transgenic lines enable detailed, in vivo imaging of cranial blood vessels and immune cells within the CNS [139,140,141].

Recent research by Gibson et al. identified a mechanism in zebrafish related to vascular damage during cryptococcosis, potentially contributing to complications like cortical infarction in cryptococcal meningitis [141]. In a comprehensive review by Rosowski et al., an overview of studies of cryptococcosis in zebrafish is presented, along with an exploration of the utility of this model for the investigation of other fungal infections [142]. 

### 3.6. 3R’s Concept

With the benefits of using vertebrate models for cryptococcosis also come rising concerns about the ethical aspects of research using animal models, the higher maintenance costs, and more elaborate experimental manipulations [48,80]. This presses on the need to adapt novel and alternative (in silico) approaches in order to minimize animal experiments where possible [143,144,145]. 

If animal work is inevitable within this context, the ethical concern should be implemented according to the 3 R’s concept introduced by Russell and Burch, in which replacement, reduction, and refinement stand central [146,147,148,149]. Replacement encompasses alternative options to the use of animal models. Even when animals are necessary for research, the number of animals should be reduced to the uttermost minimum. Refinement includes the least amount of harm, distress, suffering, and pain possible for the animals to improve their welfare. 

Studies indicate that enhanced animal well-being has a direct positive impact on advancing scientific endeavors, as the physiological and immunological responses in animals are influenced when their welfare is compromised [150,151]. In this context, zebrafish might be a potentially interesting alternative to higher-order forms of life when conducting fundamental research. The advantages and disadvantages of different animal models are summarized in Table 2.

## 4. Routes of Inoculation

When initiating an infection in an animal model, an important factor is the chosen route of inoculation. In early animal models, infection was often initiated by IPI of cryptococci with the specific aim of studying the formation and development of cryptococcomas [152,153]. However, these restricted methods have mainly disappeared from current research practice with the rise of longitudinal follow-up procedures [8,154,155,156]. The general approach has thus shifted towards studies that allow for more widespread dissemination. As the most severe clinical manifestations of a cryptococcal infection are attributed to CNS dissemination, this is also reflected by the chosen approaches [157,158]. 

The three most frequently used routes of inoculation are (i) STI, (ii) IVI, and (iii) ITI/INI [62,159,160]. These routes of inoculation diverge largely in the rate of neurological events (e.g., formation of cryptococcomas, meningoencephalitis), with ITI/INI resembling the most natural route of infection [161].

### 4.1. Stereotactic Injection

STI, also called intracranial injection, is the process in which a needle is inserted directly into the brain through a cranial puncture/bure hole [162]. Stereotactic techniques that use coordinates derived from rodent brain atlases are traditionally used to reach well-defined and often deeper regions of the brain that would otherwise be inaccessible [163,164,165]. Two variants of STI are the intracerebral injection, specifically aiming to provoke an immune reaction by administering cryptococci directly into the meningeal space [50], and the intracisternal injection, where cryptococci are administered into the cisterna magna [91]. STI in models of cryptococcosis of the CNS allows for the injection of a controlled number of fungal cells into the brain parenchyma [105]. This is of particular interest when the main aim of the research project focuses on the developmental stages of cryptococcomas in the brain. These lesions develop mostly in the thalamus, olfactory bulb, and periventricular space and are noteworthy characteristics of a cryptococcal infection of the CNS [166,167]. 

STI, with its ability to develop cryptococcomas in a controlled yet relatively fast way, is subsequently a noteworthy route of inoculation to specifically address this problem [168]. The lesion formation can be tightly controlled in time and space, and it has the advantage that the first signs of CNS infection occur mere days after the procedure. The STI method is also often used to provoke meningoencephalitis without the need for a prior systemic infection [169,170]. Despite the distinct benefits of STIs, a major drawback of this inoculation route is the questionable clinical relevance of this model. While a natural cryptococcal infection occurs initially after inhalation of fungal spores, STI completely circumvents the natural (pulmonary/hematogenous) dissemination and accompanying immune response that otherwise form the basis for the often fatal meningoencephalitis [25,171]. The STI model remains a fast and effective yet artificial way of assessing the neurological aspects of cryptococcosis. In order to delve deeper into the natural progression of disease development, alternative methods of inoculation should be considered. 

### 4.2. Intravenous Injection

To overcome the previous notion of a lack of systemic response, the IVI model is the preferred route of inoculation in order to achieve a relatively fast yet dispersed infection and successive system-wide immune reactions [93,172]. By directly administering the fungal cells into the bloodstream, this model closely mimics the dispersion stage that occurs after the initial lung infection [173,174,175]. In other words, the IVI model enables a relatively rapid and reproducible investigation of later phases of the disease that would otherwise only occur after long incubation periods in models such as the intraperitoneal route of inoculation. 

The IV route of infection permits well-controlled disease initiation and progression due to the fact that, similar to the STI model, the number of fungal cells injected can be tightly controlled [176,177]. This furthermore allows for a highly reproducible and reliable model that has already been optimized for mice and rats [92,93,94,110,178,179]. Contrary to the STI, intravenously infected animals will have a slightly longer yet still relatively fast disease incubation time before the first signs of morbidity arise [22]. This enables the researcher to discriminate degrees of virulence between cryptococcal isolates more easily when examining different transgenic strains or different molecular types within the *Cryptococcus* species complexes [176,179]. This is closely related to one of the biggest conundrums within the field, namely how relatively large fungal cells like cryptococci transverse the BBB [180,181]. The IVI model closely represents later clinical manifestations of the disease as well as the activation of the immune response in the bloodstream and the brain, barring some exceptions to the pulmonary system [182,183]. Similar to clinical and veterinarian cases, cryptococcal cells disseminate not only to the CNS but to principally all majorly vascularized organs during the terminal stages of the disease, such as, but not limited to, the liver, spleen, kidneys, and, to a lesser extent, the lungs, where cryptococcal colonies and even lesions can be identified by post-mortem histopathology [1,184].

Excluding HIV^+^ patients, another principal patient group affected by *Cryptococcus* is organ transplant recipients, whose organs were infected before implantation [185,186]. Taken together, the IVI model is ideal for studies aiming to provoke a relatively fast and widespread dissemination of cryptococcal infection, mainly towards the CNS. However, an insurmountable shortcoming of this technique is the exclusion of the pulmonary system, where the infection normally initiates. This not only skews the dissemination results but also affects the initial immune response, as pulmonary macrophages are coined as one of the prime mediators in both the containment of the infection and the dissemination, especially towards the CNS [187,188].

Finally, it is important to note that the inoculum used for IVI is traditionally orders of magnitude higher compared to the natural/environmental occurrence, resulting in potentially artificially forced dissemination and accumulation/occlusion in the blood vessels [141]. In conclusion, the IVI is a fast, reliable, and reproducible model for studies involving the CNS. Yet some of the aspects of the resulting infection are distant from what would be expected within the natural pathogenesis of the disease.

### 4.3. Intratracheal and Intranasal Installation

Pathogenesis in clinical cases usually starts after inhalation of cryptococcal cells or spores, which are abundantly present in the environment [25]. Thus, to recreate an animal model that mimics this natural infection as closely as possible, the ITI route of inoculation would be the preferred approach. There are currently two major methods to achieve this. In the first setup, the animal is introduced to a breathing chamber where it is exposed to aerosol-containing cryptococcal cells [26,51]. Despite this being the most physiologically relevant route of infection to rapidly deliver substances to the host with minimal adverse effects, it is nearly impossible to accurately assess the number of cryptococci inhaled by the animal, making the disease’s progress highly unpredictable and less reproducible. In recent years, Buxco, for example, has enhanced its nose-only inhalation systems by incorporating various features that enhance its flexibility and optimize its utility for pre-clinical research studies [189]. 

To overcome the numerous hurdles, a second model may be preferable, in which the cryptococcal cells are intranasally introduced to the animal [190]. This way, the inoculum introduced to the subject is more controlled. However, a risk of cryptococcal colonization of the nasal cavity exists [191]. Due to its relative closeness to cranial space, especially in rodents, it is possible that the nasal colonization might interfere with dissemination routes to the brain (lung–bloodstream–brain vs. nasal cavity–brain). In addition, an intranasal introduction has the disadvantage that the delivered number of cells is difficult to control as the animal may sneeze out part of it. Another way to introduce cryptococci to the lung is through intratracheal instillation by inserting a tube/syringe into the trachea. By directly transferring the cryptococci into the lung, possible contamination of the nasal cavity is avoided. As mentioned previously, the initial pulmonary immune response is regarded as one of the most essential hallmarks in the subsequent development of systemic cryptococcosis and its eventual dissemination towards the CNS. ITI may allow for future assessments of the impact of specific alveolar immune components during a longitudinal follow-up.

One of the most remarkable theories on how *Cryptococcus* crosses the BBB, called the “Trojan Horse Hypothesis”, suggests the involvement of alveolar M2 macrophages that act as carrier host cells for cryptococci in order to surpass several physiological barriers, after which the fungal cell exits its host through a process called vomocytosis [1,61,63]. This theory has yet to be proven conclusively in an in vivo study. The ITI may be a suitable method to assess the potential of this hypothesis. A second theory for BBB crossing is based on the idea that *Cryptococcus* is able to transverse the BBB through transcytosis [1]. 

Past in vitro studies have produced some evidence that cryptococcal cells can adhere to endothelial cells through interactions between the cryptococcal capsule and CD14 on the surface membrane of these cells before internalization by these border cells [192,193]. Cryptococci may furthermore be aided by the weakened state of the BBB that often occurs in immunocompromised patients. 

Another major theory for cryptococcal CNS infiltration is often referred to as the paracytosis hypothesis. CN/CG are able to produce a wide range of degradative enzymes such as urease, phospholipases, IL-13, and IL-33 [194,195,196]. These enzymes heavily affect the molecular structure of the tight junctions between the epithelial cells of the BBB. By simultaneously weakening these junctions and inhibiting the production of E-cadherin (a vital component of tight junctions), it is possible for cryptococci to enter the brain operculum [197]. 

Despite the obvious advantages of this approach over the STI and IVI routes of inoculation, the ITI model has two major disadvantages inherent in its setup. The first disadvantage is the slow dissemination process. If one uses a physiological but relatively low inoculum, the infection might be cleared before any manifestations occur. Also, the temporal profile of disease development is more variable than in the STI and IVI models. On the other hand, the use of a higher inoculum might cause the animal to die from a lung infection before the disease disseminates to the CNS. Taken together, it is crucial for the ITI to find the right inoculum concentration that balances between clearance and premature death of the animal [191]. Careful and strain-specific optimization is needed for this model in mice and rats [191,198]. A second hurdle when using this technique is the variable disease outcome and lower level of reproducibility. Nonetheless, an optimized form of the ITI model is a promising tool for future studies that aim to address the evolution of cryptococcal infections as a longitudinal process ranging from the initial pulmonary infestation up to the final stages of the disease in which neurological manifestation occurs. The advantages and disadvantages of the different forms of initiating cryptococcosis in vertebrate models are outlined in Table 3.

## 5. Evaluation of Infection

### 5.1. Ex Vivo Methodology

Collecting tissue biopsies and body fluids of organs post-mortem for further ex vivo analysis is the standard approach for confirming the presence of infectious pathogens and their interaction with the host. This is normally conducted in order to quantify cryptococcal infection in organs/tissue of interest or to assess the immunological response in regions of interest. A gold standard in microbial research is the counting of colony-forming units (CFU), also known as the clonogenic assay [199,200,201,202]. CFU counts provide a reasonable estimation of viable cryptococcal cells in a sample. This may function as a crude approximation of the fungal load in specific tissues [199]. Other frequently used methodologies are histopathological assessments and flow cytometry [31,203,204]. Both techniques provide additional and detailed immunological data for the in vivo experimental results and are also useful for post-mortem confirmation of in vivo analyses. 

Specific stainings that are applied for a more detailed analysis of CN/CG include Indian ink, Hematoxylin and Eosin, and Periodic Acid–Shiff stainings [49,205,206]. In particular, the capsule is targeted by some of these stainings [207]. Apart from the identification of cryptococcal cells, immunohistochemistry and flow cytometry are frequently performed when assessing immunological reactions in response to CN/CG presence [109,208,209]. This is particularly the case when focusing on host–pathogen interactions within the meninges and in the near cerebral lesion environment. 

### 5.2. Characterization of Animal Models of Cryptococcosis by In Vivo Imaging

While the majority of animal models are assessed by performing survival studies using invasively collected tissue/body fluid samples (blood, CSF) or acquiring post-mortem data by histopathology/immunohistochemistry [199,202,210], allowing in-depth analysis of disease manifestation, they rely on single time points and greatly interfere with our ability to understand very dynamic (patho)physiological processes that occur during host–pathogen interaction but also during treatment [211,212]. In contrast to invasive, single-time point studies, in vivo imaging opens up the opportunity to follow dynamic processes during disease progression and treatment by studying the same animal repeatedly in longitudinal experiments [149,213,214,215]. Hereby, each animal acts as its own control with data collection before inoculation (control), repeatedly during the development of the infection, and possibly during treatment. This increases statistical power (for example, identification of failed infections) and reduces the number of animals needed for preclinical studies, which potentially reduces costs and is in line with the societal quest towards a more ethical incitement on animal studies, also implemented in the 3 R’s principle (see above) [149,216].

Non-invasive imaging methods are essential for the diagnosis and management of many diseases, in particular neurological disorders. This also applies to the diagnosis and treatment assessment of brain infections. The most commonly used imaging techniques in the central nervous system include computed tomography (CT), magnetic resonance imaging (MRI), and methods using radioactive tracers like positron emission tomography (PET) and single-photon emission computed tomography (SPECT). Existing clinical imaging modalities provide rapid and reliable assessment of brain disorders; however, they are often not specific to infections of the CNS [217,218]. As animal models are a valuable tool to understand the underlying mechanisms of CNS infections, non-invasive imaging of animal models has evolved into an important research tool in the biomedical sciences. This development was, on the one hand, driven by the development of disease models and transgenic animals to better understand diseases and their treatment, and on the other hand, by the availability of imaging hardware that is suitable for imaging small animals. 

The objectives of using small-animal imaging modalities include the development and evaluation of new imaging methods for diagnostic purposes; a better understanding of the pathogenesis and pathophysiological mechanisms of diseases, in particular, their dynamic patterns; the evaluation of new therapeutic approaches and their mechanisms of action; the validation of new drugs and diagnostic tracers/contrast agents; the evaluation of transgenic organisms for their suitability to model diseases in humans; and others. Small-animal imaging has many modalities comparable to clinical imaging. The principle of the imaging methods is identical, independent of the size of the patient. Some imaging methods have to account for higher dosages of radiation or radioactive tracers per body weight when comparing small-animal imaging with clinical methods (for example, CT or PET). Optical methods are almost absent from clinical studies (except for intraoperative or endoscopic procedures) due to the usage of genetic transformations (expression of bioluminescent (BLI) or fluorescent imaging reports) and insufficient depth penetration. All imaging methods have to account for the small size of the animals, which can partly be compensated for by the development of dedicated instrumentation. In addition, attention has to be paid to animal handling and monitoring in order to work as closely as possible with physiological conditions. Some imaging methods that are rarely used for imaging of the brain, mainly due to artifacts caused by the skull, like ultrasound and photo-acoustic imaging, have been further developed for applications in the rodent brain [219]. However, they have not been used for brain infections yet. Table 4 provides a very general overview of the advantages and disadvantages of the techniques most commonly used for the in vivo characterization of animal models.

### 5.3. Optical Imaging

Optical imaging uses visual, infrared, or ultraviolet light to visualize cellular and molecular processes in vivo. Those methods are relatively cost-efficient, sensitive, and rapid when compared to other imaging techniques. There are two main types of in vivo optical imaging methods: bioluminescence imaging (BLI) and fluorescence imaging (FLI). BLI and FLI, including near-infrared fluorescence (NIRF), allow non-invasive imaging of physiology and metabolism down to the molecular level. In contrast, most multiphoton/intravital microscopy imaging approaches are (semi) invasive optical imaging techniques. For overviews of applications for BLI and FLI (for example [220,221,222,223]). 

As targeted cells and molecular processes cannot be directly imaged, both BLI and FLI require the use of optical imaging probes and/or reporter genes. BLI requires genetic alteration of cells with a reporter gene (e.g., luciferase). After injection of a substrate such as luciferin, substrate oxidation occurs, and emitted photons can be detected by a camera. FLI is able to use native, unaltered cells for the visualization of molecular events in animals. A fluorescently labeled targeting agent (peptide, protein, or particle) is injected into the animal, where it will either be cleared from the animal’s circulation over time or retained by binding it to a specific target. Upon excitation with a light source, the fluorescent dye will emit photons that are collected by a sensitive detector. Alternatively, FLI can also be used to visualize cells that are genetically labeled with a fluorescent reporter gene. 

Because BLI and FLI are optical imaging technologies based on light, they suffer from the fact that light transmission in living subjects is hampered by tissue scattering and absorption [224]. This is more important for FLI as it needs both excitation and emission photons to travel through tissue, whereas for BLI, only the emission photons are affected. Absorption of photons with wavelengths lower than 600 nm is mainly due to the presence of molecules such as melanin, hemoglobin, water, and lipids and occurs at specific wavelengths. At longer wavelengths, the scattering of light becomes a more important attenuation factor than absorption.

Bioluminescence imaging is based on the enzymatic reaction catalyzed by substrate-specific enzymes called luciferases. In the presence of oxygen and certain cofactors, such as ATP, luciferases oxidize a specific substrate and thereby release visible light photons through a chemiluminescence reaction without the need for excitation by an external light source. All luciferases have in common is that they are catalyzed reactions of molecular oxygen with a substrate that all involve a luciferase-bound peroxy-luciferin intermediate, the breakdown of which provides energy for excitation. This intermediate holds the reaction energy for no more than a few nanoseconds, after which the energy is released in the form of photons [225]. During the emission of photons, oxyluciferin is contained in the enzymatic cleft, allowing interactions with the luciferase to change the amount of energy released and, therefore, the wavelength of the emitted light. This phenomenon has been used to generate luciferases that emit light of different wavelengths by mutating crucial amino acids near the enzymatic cleft [226]. The most widely used luciferase for in vivo imaging in the rodent brain is firefly luciferase (fLuc) [227]. 

fLuc’s short half-life of about 2 h prevents its accumulation in the cytoplasm, which enables ‘real-time’ imaging once the substrate D-luciferin is applied. After IV or IP substrate administration, the substrate redistributes almost immediately to different body compartments. It is also capable of crossing the intact BBB, which is essential for imaging inside the CNS. The oxygenation of luciferin for producing a BLI signal is ATP-dependent. Therefore, fLuc BLI can be used for assessing the viability of cells.

Luciferase reporters have been integrated into bacteria, viruses, pathogenic yeasts, and parasites, enabling the assessment of infection in vivo, including its response to treatment and host–pathogen interactions [228,229,230,231,232,233,234,235]. Transgenic modification of yeast cells, expressing luciferase under a specific promoter, can, for example, be used to monitor cellular changes like the formation of hyphae during biofilm formation [236,237]. 

**Figure 2:**
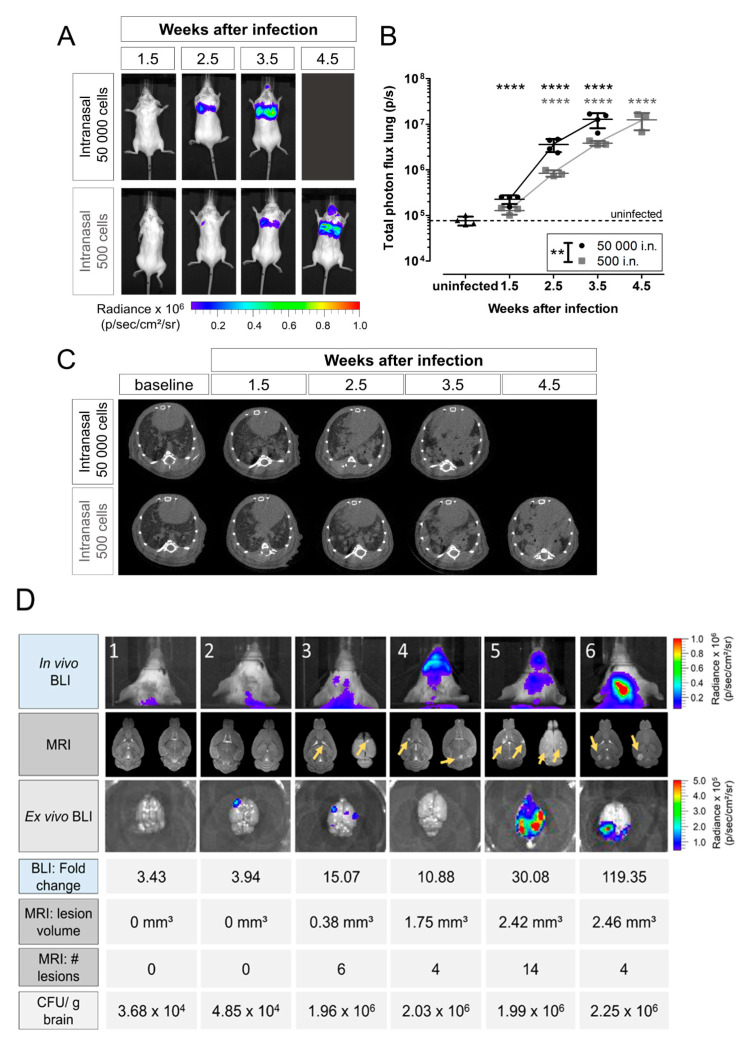
Multimodal imaging of a mouse model of cryptococcosis instilled via intranasal inoculation. (**A**) In vivo BLI showed the development of a progressive pulmonary and, to a minor extent, nasal or sinus infection upon intranasal inoculation with 50,000 or 500 firefly luciferase-expressing *C. neoformans* KN99α-CnFLuc cells. ** *p* <0.01; **** *p* < 0.0001, two-way RM ANOVA with Bonferroni post-test, comparison with uninfected controls (BlI, *n* = 4) or baseline (CT). (**B**) Quantification of the bioluminescence signal from the lung region demonstrated significantly different progressions of infection in both inoculum size groups. (**C**) Lung CT imaging confirmed dense lesion formation (gray) in the normally aerated (black) regions of the lungs. (**D**) Dissemination of the infection to the brain as illustrated by in vivo BLI, MRI, and ex vivo BLI with the corresponding values of fold change in BLI signal (compared to baseline), MRI lesion number and volume, and CFUs/g brain at week 5 showed the extent of infection for individual animals. Animals 1 and 2 (10^4^ CFUs/g brain) did not present with visual lesions in BLI or MRI but had a 3- to 4-fold increase in the in vivo BLI signal in week 5. Animals 3, 4, and 5 had multiple small lesions on MRI (arrows) and a 10- to 30-fold increased BLI signal. Animal 6 presented with one localized hotspot on BLI, corresponding to a large lesion on MRI. Modified from Figures 3 and 5 of Vanherp et al. (2019), doi:10.1242/dmm.039123, The Company of Biologists [179].

In *Cryptococcus* research, BLI has been used to quantify the viable fungal load during the progression of pulmonary and cerebral cryptococcosis, for monitoring the dissemination from the lung. to the brain (see Figure 2A,B), and for assessing therapeutic success [155,179,238]. Hereby, non-invasively determined BLI signal intensity was directly correlated with CFU counts, confirming that viable fungal load can be determined in vivo using BLI. Besides the fact that cryptococci need to be genetically modified for the expression of luciferases, BLI remains a promising tool for the evaluation of pathogenesis and possible therapeutic drug targets [239].

Fluorescent imaging relies on the emission of photons after the excitation of fluorescent probes. Some shortcomings of this technique, such as photon scattering and background fluorescence emitted by certain types of tissues, are nowadays largely solved by the creation of near-infrared fluorescent probes and the implementation of emission filters. Fluorescent imaging allows for quantitative read-outs and even 3D tomographic in vivo image reconstruction. However, sensitivity for in vivo detection remains limited. An example of in vivo and ex vivo FLI in cryptococcosis research is the CTR4-fluorescent fusion reporter to study the role of CTR4 in copper-related homeostasis and subsequently in fungal virulence [240]. Other applications include the use of fluorescent nanoparticles, as in a study conducted by Silva et al. [100]. This research centers on the creation of quantum dot conjugates utilizing lectins from schinus terebinthifolia leaves (SteLL) and punica granatum sarcotesta (PgTeL), with a specific focus on labeling CN cells. This underscores their potential as specialized nanoprobes for investigating glycobiology in *Cryptococcus* [100]. 

Moreover, various investigations aim to genetically modify cryptococcal strains for the expression of fluorescent proteins like m-Cherry. For example, Upadhya et al. conducted a study in which they utilized a ‘safe’ locus within a cryptococcal strain to generate a genetically stable transgenic KN99mCH strain that exhibited high fluorescence [241]. Fluorescent antimicrobial peptides have been used for targeting fungi (*A. fumigatus*) for real-time imaging of fungal infection [242]. While targeting efficiency was remarkably high and specific, imaging was mainly performed in situ. Similar approaches are also promising for research targeting CN and CG. Other applications relevant for *Cryptococcus* research include the targeting and in vivo imaging of macrophages by fluorescent probes in order to monitor pathogen–host cell interactions [243,244]. In addition to applications in FLI, fluorescent reporter genes as well as fluorescent probes are essential for multiphoton microscopy. 

Multiphoton/intravital in vivo microscopy is able to bridge the gap between the low resolution of purely non-invasive imaging techniques and the invasiveness of standard microscopy methods that rely on ex vivo tissue samples. Multiphoton microscopy allows the evaluation of cellular events in deep tissues in living animals in real-time settings at singular or multiple time points. As it uses a multiplex approach to being able to detect different frequencies, interactions between the host’s immune cells and pathogens can be studied. For this technique, fluorescent cells, contrast agents, or autofluorescence are utilized [245]. A conventional fluorescent microscope exploits only one photon to excite a fluorophore, whereas a multiphoton microscopy excites fluorophores with multiple photons [246]. As such, it is important to consider the appropriate modality to reach sufficient depth to image cells and tissues of interest [247]. Multiphoton microscopy can be used when the target is located near the surface of the targeted tissue [223,247]. It often uses near-infrared (NIR; 700–1700 nm) excitation wavelengths, which can excite fluorescent dyes by absorption of two (or three) photons (2P) in the NIR range, of which the combination of the energy of these photons in each excitation is larger than the gap between ground state and excited state [223,247,248]. 

The relationship of 2P-IVM is non-linear, more specifically quadratic, meaning that there is a high density of photons at the focal point, causing localized excitation and, thus, high spatial resolution [249]. Additionally, NIR imaging employs higher wavelengths with lower energy, which is preferable for deeper penetration due to less scattering. Therefore, much greater depths can be reached with 2P-IVM compared to conventional confocal microscopy, which displays more scattering [249,250,251]. As there is also no out-of-focus fluorescence in 2P-IVM, the scattered light can be assigned to the field of view (FOV), resulting in greater signal intensity [250,251]. For in vivo applications, either a sufficiently transparent organism is needed, like in studies using zebrafish [252] or through implanted transparent window chambers (cranial window, lung window, etc.) and small incisions in the skin when using rodents [253]. 

In the realm of fungal infections affecting the brain or meninges, some of the primary culprits include *C. albicans*, *A. fumigatus*, and CN/CG [1,6]. The latter is known for causing cerebral and meningeal infections [254]. In order to infiltrate the CNS, cryptococci must traverse the BBB, a process that can be evaluated using MP-IVM. Two previous studies performed by Zhang et al. and Shi et al. used IVM in order to elucidate mechanisms of cryptococci entering the CNS, in which both research groups visualized the interaction of the host and the pathogen [255,256]. 

Using intravital microscopy, Zhang et al. illustrated the migration of neutrophils towards cryptococci in the brain microvasculature, a primary culprit in causing meningoencephalitis [256]. Through the utilization of intravital microscopy, Shi et al. have demonstrated that CN undergoes abrupt cessation within mouse brain capillaries possessing a diameter equivalent to or smaller than the organism [256].

A similar approach using a semi-invasive procedure to detect fluorescently labeled pathogens uses fiber-optical probes. Fibered confocal fluorescence microscopy (FCFM) was used to study pulmonary and CNS fungal infections in rodents, thereby achieving cellular resolution for the assessment of cell densities at sites of infection [191]. This approach can be used repeatedly on the same animal without causing major tissue damage. In a model of cerebral cryptococcosis, FCFM was used in combination with parametric MRI to confirm capsule size differences in vivo [36].

### 5.4. Computed Tomography

Computed tomography (CT) is based on the absorption of X-rays by different tissue types, thereby generating contrast based on differences in tissue density (air, soft tissue, and bone). The principle of CT was introduced in the mid-1960s by Sir Godfrey Hounsfield, who proposed to reconstruct a 3D object from a series of 2D projections using filtered back projection, which resulted in the first clinically used CT scanners by the beginning of the 1970s [257,258]. CT is probably the most used method in clinical radiology due to its cost-efficiency and availability. Its advantages are the very high resolution that can be achieved and the excellent contrast for tissues with large density differences (for example, bone to air or bone to soft tissue). Without the utilization of contrast agents, soft tissue contrast is rather low but can be improved by using heavy-atom contrast agents, for example, for CT angiography. Dedicated, high-resolution small-animal CT scanners have been commercially available for many years [259]. 

CT is the method of choice for imaging the lungs, which is particularly important for the diagnosis of pulmonary infections like in the case of cryptococcosis [260,261,262]. Based on how well defined the border regions are, multiple classes of lesions can be identified, ranging from well-delineated nodules to more vague miliary patterns [261,263]. Semi-quantification of CT data acquired over a time span may help in assessing aspects of cryptococcosis, ranging from virulence to lesion development and changes in lung volume over time [264]. For the characterization of pulmonary cryptococcosis models, CT protocols have been developed for quantification of lesion formation and lung parameters like total lung volume and aerated lung volume, which can be used to monitor disease progression and potentially therapy [179,234,265]. Figure 2C illustrates the use of preclinical CT in a multimodal approach, combining CT, BLI, and MRI to study the temporal evolution from an initial pulmonary infection to the dissemination to the brain [179]. While the use of ionizing radiation in CT imaging is a potential concern, in particular for repeated follow-up imaging [266,267], no radiotoxicity was found in carefully designed longitudinal studies [268]. In addition to dedicated applications in pulmonary infectious disease models, CT also provides an anatomical reference frame for imaging modalities that lack anatomical information, like PET and SPECT.

### 5.5. Radionuclide Imaging 

PET and SPECT imaging both rely on radioactive tracers that either target specific transporters or receptors or form substrates for specific metabolic pathways. PET uses tracers that emit positrons (^18^F, ^11^C, ^89^Zr, etc.). SPECT uses tracers that emit gamma rays or high-energy X-ray photons (^99^mTc, ^123^I, ^133^Xe, etc.). These radioactive tracers have half-lives ranging from a few minutes up to several weeks. Similar to optical imaging, contrast in PET and SPECT images is highly specific, which often results in limited anatomical background information, so modern PET and SPECT scanners are combined with CT or less frequently with MRI scanners [269,270,271]. The main advantages of PET and SPECT over other in vivo imaging techniques are their high sensitivity, which allows for tracer concentrations well below physiological ranges (nM to pM range), and the direct targeting of molecular processes like receptor-ligand or transporter binding, which makes them true molecular imaging methods. The major disadvantage of PET and SPECT is their low spatial resolution, in particular for applications in rodents. As a glucose analog, FDG is transported into cells via glucose transporters. It is phosphorylated in the glycolytic pathway. After phosphorylation, FDG cannot be further metabolized and is trapped in most cells. Therefore, accumulation and retention are directly linked to rates of glycolysis. FDG-PET is widely used for the diagnosis and assessment of malignancies and in preclinical tumor models, but also in inflammatory processes [263,272,273,274]. The high specificity of FDG-PET to a metabolic pathway like glycolysis paradoxically also contributes to its unspecificity, as glycolysis is common to all cells. 

This is also reflected in clinical case reports and reviews on cryptococcosis and other fungal infections, where FDG-PET was used to distinguish between malignancies and cryptococcoma [275,276]. The increased uptake of FDG in cryptococcal lesions is most likely due to a combined effect of inflammation and uptake by cryptococci. Similar to FLI, successful experiments have been performed to label antifungal compounds or fungus-specific antibodies with radioisotopes in order to study their biodistribution or use them as theranostic agents [275,277,278,279]. Besides some early success, no larger-scale studies have been performed on animal models of cryptococcosis. The high potential of immune-PET for pathogen-specific diagnosis and therapy follow-up was, however, demonstrated in models of pulmonary aspergillosis [280]. 

### 5.6. Magnetic Resonance Imaging and Spectroscopy 

**Figure 3:**
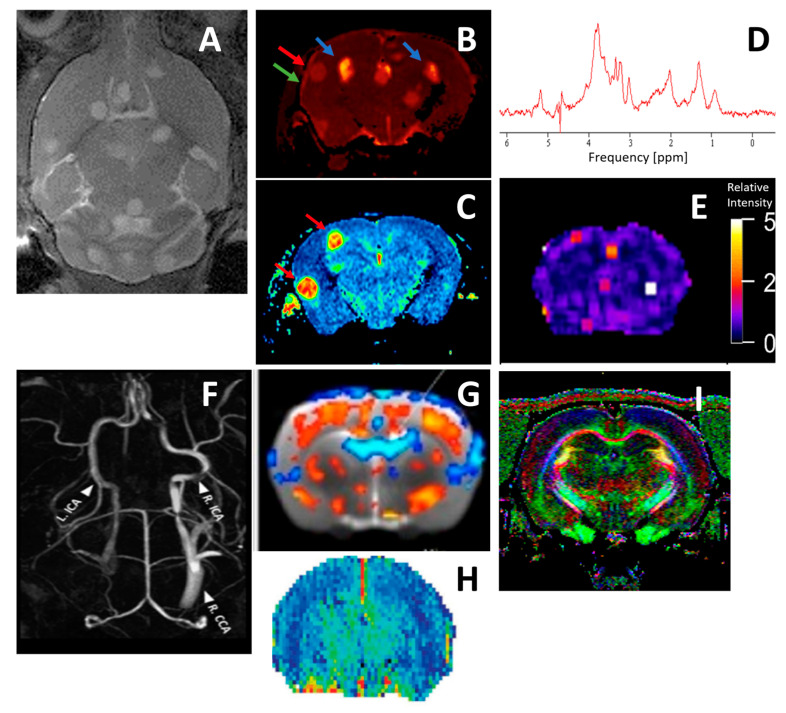
Illustration of some different MRI protocols that contribute to multiparametric, quantitative readouts. (**A**–**E**) Mouse brains that were infected with CN by intravenous injection. (**A**) anatomical T2-weighted MRI, illustrating hyperintense lesions. (**B**) parametric T2 map (arrows indicate regions of increased T2 values: blue – lesions, red and green – meningitis), (**C**) parametric map of apparent diffusion coefficients, (**D**) MR spectrum of a lesion that is dominated by trehalose, mannitol, and lipid signals, and (**E**) CEST spectrum, illustrating hyperintense signals due to trehalose content in small lesions. (**F**) MR angiogram, illustrating vascular remodeling after occlusion of the left CCA, (**G**) resting state fMRI, illustrating regions of increased (red) and decreased (blue) neural activity, (**H**) quantitative cerebral blood flow map, and (**I**) diffusion image, illustrating anisotropy in diffusion (for example, along the corpus callosum).

Magnetic resonance imaging (MRI) has developed into one of the most powerful imaging tools, not only in radiology but also in preclinical research. The excellent soft tissue contrast with high spatial resolution (see Table 4) makes it the method of choice for anatomical, functional, and molecular imaging of the brain [281,282,283]. MRI is based on the manipulation of the magnetic properties of ‘MR-active’ nuclei (for example, ^1^H, ^13^C, ^19^F, ^31^P, etc.), of which hydrogen is the most abundant, most sensitive, and most used atom, also because it is ubiquitously present in living organisms in the form of water and lipids. MRI requires a homogenic static magnetic field to generate different energetic states. Using a radiofrequency pulse, the energetic states of the nuclei are modulated. Protocols using different trains of radiofrequency pulses (so-called pulse sequences) are applied to make use of different contrast mechanisms. Hereby, various endogenous properties of the tissue as well as exogenous agents can be utilized. In particular, in neurological research, high-resolution MRI provides not only information on anatomy/morphology but also on hemodynamics (blood flow, blood volume, and tissue perfusion) [284], metabolic changes (MR spectroscopy) [285], brain function (functional MRI, fMRI) [286], and functional connectivity (diffusion imaging, manganese-enhanced MRI, and MEMRI). The availability of responsive and targeted contrast agents extends applications of MRI from the visualization of cell location to the characterization of molecular and cellular signaling events [287]. An example of the multi-parametric approach that can be taken by MR imaging and MR spectroscopy is shown in Figure 3.

Applications of MRI in preclinical research on cryptococcosis are still relatively sparse. MRI cannot only be used to study dissemination to the CNS by detecting and quantifying lesion formation but also to validate the success of therapy, in particular when combined with other imaging modalities like BLI [155,179]. Using multiparametric maps, like the determination of apparent diffusion coefficients or relaxation times, allows for the quantification of water mobility and hereby assesses cell densities. This was used to compare the relative cell densities of lesions formed by CN and CG and, indirectly, the size of their capsules in vivo [36]. As the differential diagnosis of cerebral cryptococcoma from cystic gliomblastoma and brain abscesses based on radiological findings remains challenging [105,288], it is important to identify diagnostic markers.

Trehalose and mannitol are compounds that are not produced in high concentrations in mammalian tissue or abscess-causing microorganisms but by CN and CG. Trehalose can be identified by in vivo MR spectroscopy in animal models and in patients with large-sized cryptococcoma [168]. The use of trehalose as a marker compound has been further optimized in so-called CEST MRI protocols, which also allow for the identification and quantification of trehalose in small lesions [289]. The in vivo quantification of trehalose is not only useful for clinical diagnosis but also for the follow-up of therapy. This is in particular essential for patients under long-term treatment who suffer from side effects. Trehalose concentrations correlate with CFU counts in in vivo MR spectroscopy experiments [168].

## 6. Discussion and Conclusions

Cryptococcosis in humans remains an active healthcare problem. The World Health Organization classification of fungal pathogens lists CN/CG as a top priority due to (i) its manifestation in immunocompromised patients, (ii) overall increased resistance towards anti-fungal therapies, and (iii) the still poor understanding of the underlying biological and immunological factors that drive cryptococcosis. While most cryptococcal infections manifest themselves in the lungs, there is a substantial risk of life-threatening neurological sequelae if left untreated. In addition, the treatment efficiency of cryptococcosis is relatively poor and has severe side effects on patients. These facts, combined with the use of *Cryptococcus* as a model organism as a general fungal pathogen, advocate for the need to better understand the underlying mechanisms that allow an opportunistic pathogen to cause such detrimental health effects in immunocompromised patients. This review aimed to provide an overview of the current state of experimental preclinical vertebrate models, in particular for CNS infections, as well as address shortcomings that are inherent to these models and their characterization. To date, three major routes of infection (stereotactic, intravenous, and intratracheal instillation) and their variations are currently used in the majority of research on cryptococcosis. The preference for one of these routes of infection is mostly based on the research questions that are addressed, often ignoring the shortcomings of the used model. While all have obvious advantages and limitations, the preference for one over others is mostly based on the ease of the model, its reproducibility, and the difference in the rate at which the disease manifests itself, in contrast to how closely the method mimics the natural infection. Taken together, most of the current research relevant to cryptococcal host–pathogen interactions has implemented either STI or IVI as the principal infection model. ITI, though closely mimicking the natural route of infection, is only used in a few studies that address CNS dissemination. Optimization of this method might lead to new insights on mechanisms by which CN/CG manage to disseminate to the CNS. 

Traditional medical mycological research using animal models was mainly centered around post-mortem single time point analysis in the past. However, with the increased availability of preclinical imaging infrastructure, there is an increased use of different preclinical imaging methods in infectious diseases research, which is already more established in preclinical models of neurological, oncological, or cardiac diseases. This is in particular important for longitudinal monitoring of cryptococcosis models, host–pathogen interactions, and the assessment of novel therapeutic approaches. Compared to invasive, long-time gold standard methods in microbiology and infectious diseases research, in vivo imaging methods have the advantage that (1) they address societal ethical concerns of research involving animal models by reducing the number of animals substantially, (2) they increase the statistical power of animal models as each animal acts as its own control (several time points, including pre-infection, inoculation, progression of infection, and potential follow-up of therapy), and (3) they are in most cases less labor intensive and potentially more cost efficient than conventionally used invasive methods. The most commonly used in vivo imaging methods include BLI for quantifying viable cells. CT is most frequently used to assess anatomical changes, in particular for lung diseases and lung infections [266,269]. PET and SPECT imaging evolve for the assessment of molecular processes using tracers for specific microbial metabolic processes or transporters. Fluorescence imaging, including intravital multiphoton microscopy, provides cellular resolution, and its multiplex (multicolor) approach is perfectly suited to monitor interactions between different cell populations. MRI/MRS is the method of choice for the study of neurological diseases. Multiparametric readouts of MRI not only allow for the assessment and quantification of structural/anatomical changes but also for the study of function, metabolism, or blood flow. In order to overcome specific limitations of individual imaging techniques (for example, in terms of resolution, sensitivity, specificity, etc.), the combination of different imaging techniques in multimodal approaches becomes more and more popular. In this regard, a multifaceted strategy may overcome current hurdles faced when attempting to characterize the often subtle yet defining divergencies between different cryptococcal molecular types. Potential future insights into underlying pathological mechanisms, immunological interactions, dissemination pathways, (neurological) manifestations, and the validation of novel therapeutic approaches that are gained by using non-invasive assessment methods may bring cryptococcal research into the modern age of personalized and case-specific medicine. In terms of translation to the clinic, in vivo multimodal/multiparametric imaging will further improve early diagnosis and, more importantly, non-invasive assessment of therapy. 

## Figures and Tables

**Table 1:** Selection of studies that were using cryptococcosis models in rodents.

Model	Species	Purpose of the Study	RoI	Reference
Mouse	CN	Immunology	IVI	Nishimura et al. [92]
				Aguirre K et al. [93]
			IVI/ITI	Zaragoza et al. [94]
		Model development	ITI	Davis et al. [95]
			ITI	Hester et al [96]
			ITI/IPI	Lim et al. [97]
		Pathogenesis	INI	Coelho et al. [62]
			FPI	Irokanulo et al. [98]
	CN/CG	Pathogenesis	ITI	Ngamskulrungroj et al. [99]
		Model development	ITI	Davis et al. [95]
			STI	Vanherp et al. [36]
	CG	Immunology	ITI	da Silva-Junior [100]
		Model development		Diniz-Lima et al. [101]
		Drug development		Oliveira-Brito et al. [102]
Rat	CN	Immunology	ITI	Rubinstein et al. [103]
				Sotomayor et al. [104]
		Model development	STI	Himmelreich et al. [105]
			ITI	Himmelreich et al. [106]
		Pathogenesis	SCI	Graybill et al. [107]
		Drug development	IVI	Alves et al. [108]
	CG	Model development	ITI	Krockenberger et al. [109]

Overview of different rodent models with respect to the used cryptococcal species, the general purpose/aim of the study, route of infection (RoI), and respective references. Infection was achieved through either intravenous inoculation (IVI), intratracheal/lung inoculation (ITI), intraperitoneal inoculation (IPI), intranasal inoculation (INI), foot-pat inoculation (FPI), or stereotactical inoculation (STI).

**Table 2:** Overview of advantages and disadvantages of different vertebrate models in cryptococcosis research and route of inoculation.

Animal Model	Advantages	Disadvantages
Mouse	- Diverse genetic backgrounds (inbred and transgenic mouse strains) - Cost-effectiveness in procurement and housing - Infection induced through different inoculation routes - Relative low maintenance costs	- Differences in susceptibility, survival rates, and inoculum doses between inbred strains
Rat	- Larger size allows for more manipulation - Natural prevalence and natural resistance of the disease	- Increased cost - Sparsity of genetically modified variants
Guinea pig	- Cutaneous fungal infection mode - Tame and medium size - Susceptible for fungal infections	- Susceptible to stress - Higher costs
Rabbit	- Well-established model for pharmaceutical/therapeutical research - Larger size allows for more manipulations	- Higher costs - Resistance to cryptococcal infection
Zebrafish	- Low costs - Fewer ethical concerns - Transgenic lines available - Optical transparency	- Compromise between invertebrate simplicity and mammalian organ complexity

**Table 3:** Overview of advantages and disadvantages of different routes of inoculation in vertebrate models of cryptococcosis. Abbreviations: stereotactic injection (STI), intravenous inoculation (IVI), intratracheal inoculation (ITI), and intranasal inoculation (INI).

Inoculation Route	Advantages	Disadvantages
STI	- Controlled number of fungal cells - Lesion formation occurs shortly (a few days) after injection	- Invasive - Limited clinical relevance - Circumvents the natural dissemination
IVI	- Enables a relatively rapid and reproducible investigation of later phases of the disease - High reproducibility	- High inoculation dose compared to environmental occurrence - Artificially forced dissemination
ITI/INI	- Models the natural route of infection as closely as possible	- Slow dissemination - Optimization of inoculation dose necessary - Lower reproducibility

**Table 4:** Key characteristics, advantages, and disadvantages of main in vivo imaging methods used in preclinical research.

Method	Spatial Resolution	Sensitivity	Specificity	Main Advantages	Main Disadvantages
MRI	<0.1 mm	−	+/−	High, soft tissue contrast Multi-parametric Functional and metabolic	Low sensitivity
PET/SPECT	1 mm	+	+	Metabolic imaging High sensitivity	Limited half-life of radioactive tracers Low spatial resolution
CT	3–50 µm	+/−	−	High spatial resolution	Ionizing radiation Low soft tissue contrast
Ultrasound	<1 mm	+	−	High temporal resolution	Artifacts (bone, air)
BLI	>1 mm	−	+	Cell viability	Transgenic organism Limited depth penetration
Fluorescence	1 mm	−	+	Multiplex imaging	Limited depth penetration
Multiphoton microscopy	<1 µm	+	+	High spatial resolution Multiplex imaging	Limited depth penetration Invasive procedures

## Data Availability

Not applicable.

## Abbreviations

2P	Two photons
BBB	Blood–brain barrier
BLI	Bioluminescent imaging
CFU	Colony-forming units
CG	*Cryptococcus gattii*
CME	Cryptococcal meningoencephalitis
CN	*Cryptococcus neoformans*
CNS	Central nervous system
CSF	Cerebrospinal fluid
CT	Computed tomography
FCFM	Fibered confocal fluorescence microscopy
FLI	Fluorescent imaging
FOV	Field of view
ICI	Intracisternal injection
INI	Intranasal inhalation
ITI	Intratracheal infection
IVI	Intravenous injection
IVM	Intravital microscopy
MRI	Magnetic resonance imaging
NIRF	Near infrared fluorescence
PET	Positron emission tomography
SPECT	Single-photon emission computed tomography
STI	Stereotactic injection
